# Easy to use and reliable technique for online dissolved oxygen tension measurement in shake flasks using infrared fluorescent oxygen-sensitive nanoparticles

**DOI:** 10.1186/s12934-016-0444-4

**Published:** 2016-02-24

**Authors:** David Flitsch, Tobias Ladner, Mihaly Lukacs, Jochen Büchs

**Affiliations:** Biochemical Engineering, AVT-Aachener Verfahrenstechnik, RWTH Aachen University, Worringerweg 1, 52074 Aachen, Germany

**Keywords:** Online monitoring, Dissolved oxygen tension, Oxygen-sensitive nanoparticles, Optical measurement, Shake flask, RAMOS

## Abstract

**Background:**

Despite the progressive miniaturization of bioreactors for screening purposes, shake flasks are still widespread in biotechnological laboratories and industry as cultivation vessels. Shake flasks are often applied as the first or second step in applications such as strain screening or media optimization. Thus, there are ongoing efforts to develop online measurement techniques for shake flasks, to gain as much information as possible about the cultured microbial system. Since dissolved oxygen tension (DOT) is a key experimental parameter, its accurate determination during the course of experiment is critical. Some of the available DOT measurement techniques can lead to erroneous measurements or are very difficult to handle. A reliable and easy to use DOT measurement system, based on suspended oxygen-sensitive nanoparticles, is presented in this work.

**Results:**

In a cultivation of *Kluyveromyces lactis*, a new DOT measurement technique via suspended oxygen-sensitive nanoparticles was compared with the conventional DOT measurement via fixed sensor spots. These experiments revealed the main disadvantage of applying sensor spots. With further cultivations of *Escherichia coli* and *Hansenula polymorpha,* the new measurement technique was successfully validated. In combination with a RAMOS device, k_L_a values were determined during the presented cultivations. The determined k_L_a values are in good agreement with a correlation recently found in the literature.

**Conclusions:**

The presented DOT measurement technique via suspended oxygen-sensitive nanoparticles in shake flasks turned out to be easy to use, robust and reliable under all applied combinations of shaking frequencies and filling volumes. Its applicability as an online monitoring system for cultivations was shown by means of four examples. Additionally, in combination with a RAMOS device, the possibility of experimental k_L_a determination was successfully demonstrated.

**Electronic supplementary material:**

The online version of this article (doi:10.1186/s12934-016-0444-4) contains supplementary material, which is available to authorized users.

## Background

Shake flasks are one of the most important types of cultivation vessel [1–3] and are widely used in biotechnological laboratories and industry [4–6]. To obtain better control and understanding of shake flask cultivations, online monitoring of important process parameters is essential. Such process parameters are the oxygen transfer rate (OTR), carbon dioxide transfer rate (CTR), respiratory coefficient (RQ), pH and dissolved oxygen tension (DOT) [7–10].

OTR, CTR and RQ are accessible in shake flasks by means of the RAMOS technology (Respiration Activity MOnitoring System, HiTec Zang GmbH, Herzogenrath, Germany and Adolf Kühner AG, Birsfelden, Switzerland) [11–13]. This technology is based on oxygen and pressure measurements in the head space of modified 250 mL shake flasks during phases of air supply and phases of terminated air supply. In the latter phase the linear decrease of the oxygen partial pressure is evaluated and from this information the OTR is calculated.

Due to the implementation of a standard autoclavable pH probe immersed into the bulk liquid, online pH measurement in shake flasks became feasible [14]. Following the same approach, online DOT measurement became possible by mounting a classical electrochemical Clark electrode into the shake flask [15, 16]. However, it has been shown that the baffling effect of these immersed electrodes can lead to significant hydrodynamic changes in shaken cultures [17, 18].

To realize a more suitable non-invasive approach for pH measurement, optical techniques based on fluorescence and pH sensor spots (optodes) were invented [19, 20]. These pH optodes have already been successfully combined with the RAMOS technology [21]. Also for DOT measurements, non-invasive optical approaches based on the oxygen-dependent quenching behavior of certain fluorescent dyes were introduced [22, 23] and applied [24, 25]. The utilized fluorescence optodes allowed convenient DOT monitoring in shake flask cultures for low shaking frequencies and high filling volumes [26, 27]. These conditions ensure that the optodes are permanently covered with cultivation broth. Unfortunately, this is not the case for cultivations with a high oxygen demand. For these cultivations, high shaking frequencies and low filling volumes are required. It was shown that, under these conditions, there is no location within a shake flask which is permanently covered with bulk liquid [28]. This leads to errors in DOT measurement. A mixed signal of the oxygen partial pressure of the gas in the headspace of the shake flask and the DOT of the cultivation broth is recorded [18]. By using a rotating optical sensor tip, which is permanently immersed in the bulk liquid without affecting the hydrodynamics, a difficult to handle but reliable DOT measurement technique is provided [18].

Oxygen-sensitive nanoparticles (with a hydrophilic vinylpyrrolidone shell and hydrophobic polystyrene core), which contain an oxygen-dependent infrared fluorescent dye [cyclometalated iridium(III) complex] were presented to be used instead of fixed optodes by Borisov et al. [22]. Recently, the biocompatibility and applicability of commercially available oxygen-sensitive nanoparticles for online DOT monitoring were successfully shown in microtiter plates [29]. The aim of the present work is to transfer this non-invasive measurement technique to shake flask cultivations with the fundamental benefit of providing a fully functional DOT measuring technique independent of the applied shaking frequency and filling volume.

## Results and discussion

### Characterization of the DOT measurement system

**Fig. 1:**
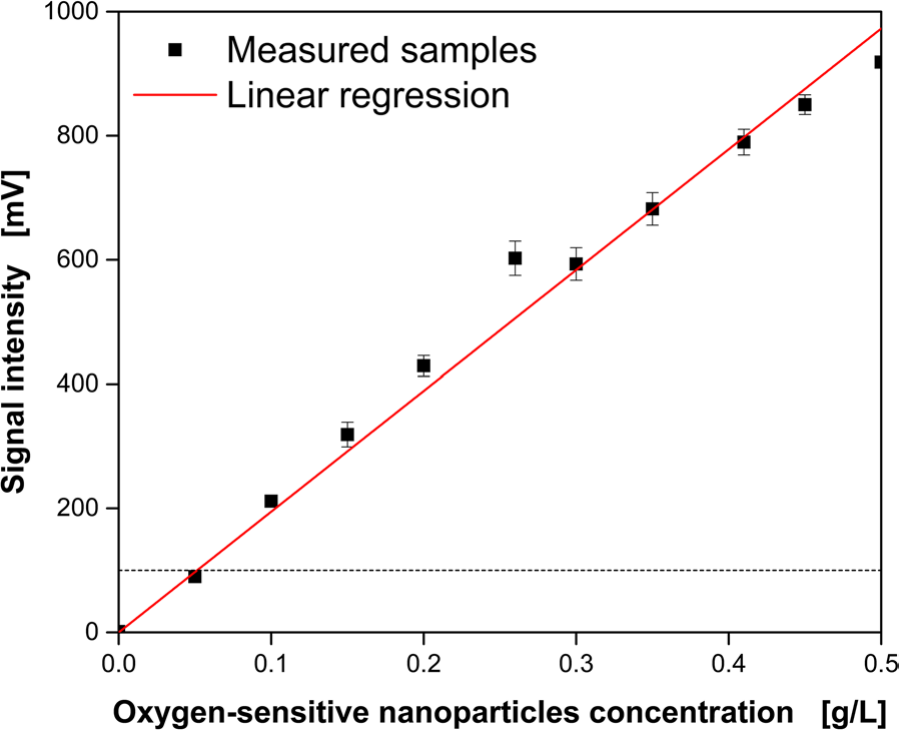
Influence of the dispersed oxygen-sensitive nanoparticle concentration on the measured signal intensity. Shown are the mean values of the determined signal intensities with corresponding standard deviations of 20 independent measurements. The *black dashed line* indicates the recommended signal intensity (100 mV) for reliable dissolved oxygen tension (DOT) measurements. Measurement conditions: Non-inoculated YM medium with varying oxygen-sensitive nanoparticle concentrations, 250 mL shake flask, 30 °C, n = 200 rpm, V_L_ = 20 mL, shaking diameter d_0_ = 50 mm

Ladner et al. [29] demonstrated the biocompatibility of the applied oxygen-sensitive nanoparticles in cultivations of *Gluconobacter oxydans*, *Hansenula polymorpha* and *Escherichia coli* with concentrations up to 1 g L^−1^. In the present study, all oxygen-sensitive nanoparticle concentrations are below 1 g L^−1^. A minimal fluorescence intensity of 100 mV is recommended by the manufacturer to obtain a reliable DOT measurement [29]. Figure 1 shows the dependency of the measured fluorescence signal intensity on the utilized oxygen-sensitive nanoparticle concentrations. The regression indicates a mainly linear correlation. The slight deviation of the proposed linear trend starting at roughly 0.25 g L^−1^ may be explained by shadowing effects of the excitation light due to a larger density of particles at higher concentrations and imperfectly linear detection characteristics of the applied photodiode. The dashed line illustrates the recommended minimal signal intensity (100 mV). Based on these results, an oxygen-sensitive nanoparticle concentration of 0.1 g L^−1^ was applied for all flowing cultivations. This choice secured sufficient signal intensities for reliable DOT measurements and kept the costs in an acceptable range.

### Oxygen-limited cultivation of *K. lactis* GG799 pKlac1 with one initial carbon source

**Fig. 2:**
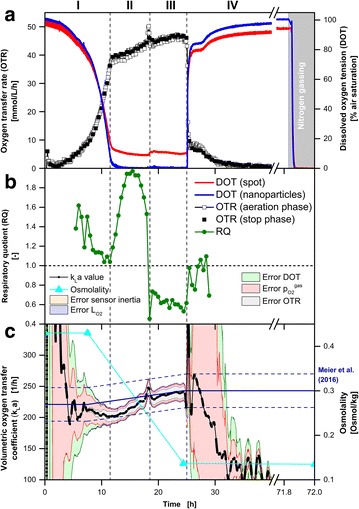
Comparison of DOT measurements via oxygen-sensitive nanoparticles and sensor spot (optode) during *K.* *lactis* GG799 pKlac1 cultivation. **a** Simultaneous online monitoring of the oxygen transfer rate (OTR) measured with a RAMOS device and the dissolved oxygen tension (DOT) measured via dispersed oxygen-sensitive nanoparticles and sensor spot in the same RAMOS shake flask. **b** Respiratory quotient. **c** Calculation (Eq. 1) and error estimation (Eq. 2) of the k_L_a values during RAMOS aeration phases. The total error is represented by the surrounding area, while the fraction of each error component is indicated by a *different color* as explained in the text. For comparison, the k_L_a value at the beginning (k_L_a_Meier,start_ = 221 h^−1^) and the end of the cultivation (k_L_a_Meier,end_ = 243 h^−1^) estimated according to the correlation of Meier et al. [34] are shown by the *solid and dashed* (±27 h^−1^) *dark blue lines*. The measured osmolality for the calculation of k_L_a_Meier_ is shown in *orange*. Cultivation conditions: Complex YEP medium with 40 g L^−1^ glucose and 0.1 g L^−1^ oxygen-sensitive nanoparticles, 250 mL RAMOS shake flask, 30 °C, n = 200 rpm, V_L_ = 10 mL, shaking diameter d_0_ = 50 mm

Figure 2 shows the oxygen-limited cultivation of *K. lactis* GG799 pKlac1 on glucose as the initial carbon source. Based on the online OTR and DOT, the cultivation can be divided into four phases: Characteristic exponential growth (I: 0–11.5 h) until the cultivation entered oxygen limitation (II: 11.5–18.7 h; III: 18.7–25 h) followed by the phase of decreasing respiratory activity (IV: 25–40 h). Oxygen limitation is indicated in the DOT measured with oxygen-sensitive nanoparticles (blue line) by reaching an air saturation close to 0 % after 11.5 h. In the OTR, oxygen limitation is usually indicated by a characteristic horizontal plateau [11, 12]. However, in this cultivation a linear increase of the OTR was detected during oxygen limitation (II, III). This can be attributed to an increase of the volumetric oxygen transfer coefficient (k_L_a) value due to the consumption of the carbon source. In general, a lower concentration of media components results in a reduced osmolality (Fig. 2c) and finally leads to increased oxygen solubility and diffusivity. Thus, oxygen could be transferred at a higher rate from the gas phase into the liquid phase (increasing OTR_max_). Within the period of oxygen limitation (after 18.7 h), a metabolic switch to a different carbon source divided the two oxygen limited phases (II, III). The metabolic switch is distinct in RQ by a steep decrease from about 1.6–0.6 (Fig. 2b). It is known from the literature that *K. lactis* produces ethanol under oxygen-limited conditions while metabolizing glucose [30, 31]. The RQ of roughly 0.6 during phase (III) is in very good agreement with the calculated RQ of the growth on ethanol of 0.625 taking biomass formation into account (Y_X/S_ = 0.2 g_biomass_ g_ethanol_^−1^). After 25 h, the initial (glucose) and generated (ethanol) carbon source were depleted and the phase of decreasing respiratory activity (IV) was initiated.

In contrast to the DOT measured via oxygen-sensitive nanoparticles, DOT measured via an optode was constantly above 8 % air saturation during the period of oxygen limitation (II, III). The expected DOT during oxygen limitation is very close to 0 % air saturation. But, by means of the fixed sensor spot constantly more than 8 % air saturation was measured. An erroneous calibration could be an explanation. To exclude this, DOT measurements were performed during nitrogen gassing after 71.8 h (Fig. 2a). Due to nitrogen gassing, oxygen is driven out of the cultivation broth and the surrounding headspace and, thus, oxygen is not present either in the gas or liquid phase. Since the DOT measured by the fixed sensor spot was also precisely at 0 % air saturation during nitrogen gassing, wrong calibration of the optode can be excluded as an explanation for the signal offset. This suggests that a mixed signal between the oxygen in the headspace of the shake flask and the DOT of the bulk liquid was measured via optode during oxygen limitation (II, III). This phenomenon has already been investigated and described by Hansen et al. [18]. It excludes fixed sensor spots for reliable DOT measurements in shake flask cultivations at cultivation conditions required for microorganisms with high oxygen demand.

Figure 2c shows the calculated k_L_a values and their corresponding uncertainties according to Eqs. 1 and 2. The fraction of each error on the total error is indicated by a different color. The applied error propagation is highly sensitive to the measured OTR and DOT, in particular for small values (Eq. 2). Since the respiration activity at the start and end of cultivations is low, the determined k_L_a values are fraught with large uncertainties which are indicated by the large errors in Fig. 2c. Therefore, the error characteristic could be divided with respect to the OTR signal into two cases. The fraction of the systematic error of $p_{{O_{2} }}^{gas}$(light red) was prevalent at low OTR values (<15 mmol L^−1^ h^−1^). The error due to the DOT measurement (light green) showed a similar but attenuated behavior. Within the range of low OTR values (0–15 mmol L^−1^ h^−1^), the systematic OTR error (grey) propagated marginally on the k_L_a value. At OTR values above 15 mmol L^−1^ h^−1^ this error fraction had a higher impact on the estimated k_L_a value. The influence of the sensor inertia error (orange) became visible after abrupt changes of the oxygen partial pressure in the headspace and the DOT [e.g. beginning of the phase of decreasing respiratory activity (IV: 25 h)]. The chemical oxygen sensor in the headspace has a longer response time (response time <15 s, [32]) than optically based oxygen-sensitive nanoparticle DOT measurement (real-time measurement, [33]). Compared to the other considered systematic errors, the error of $L_{{O_{2} }}$ (light purple) had only negligible effects on the determined k_L_a value.

In summary, the highest overall accuracy of the k_L_a value occurred during the period of oxygen limitation (II, III) due to the elevated OTR values. The most significant error in these phases was the error of the OTR measurement.

To compare the determined k_L_a values, a k_L_a calculation according to Meier et al. [34] is shown (Fig. 2c, dark blue line). Besides shaking frequency, flask size, shaking diameter and filling volume, osmolality is a key parameter for this empirical k_L_a estimation of Meier et al. [34]. Since the utilized YEP medium was not investigated by Meier et al. [34] offline samples (cyan) were taken and the required osmolality of this medium was analyzed. A linear decrease of osmolality was assumed during phases (II) and (III). At the cultivation start and after 7.5 h an osmolality of 0.43 Osmol kg^−1^ was determined, resulting in a theoretical k_L_a_Meier,start_ of 221 ± 27 h^−1^ according to the correlation of Meier et al. [34]. At this time, the corresponding experimentally determined k_L_a_exp,start_ value based on the obtained online DOT and OTR online signals was 212 ± 36 h^−1^. Both values are in good agreement within their uncertainties. The correspondi ng values after 24.4 h were k_L_a_Meier,end_ = 243 ± 27 h^−1^ and k_L_a_exp,end_ = 242 ± 15 h^−1^. These values are also well consistent within their uncertainties. A summary of all determined k_L_a values for each cultivation presented in this work is given in Table 1.

**Table 1:** Summary of experimental determined and literature k_L_a values

Strain	Medium	Culture conditions	k_L_a_exp_ (h^−1^)	k_L_a_Meier_ (h^−1^)	Osmolality (Osmol/kg)
*K.* *lactis* GG799 pKlac1	YEP medium, 40 g L^−1^ glucose	30 °C, V_L_ = 10 mL, n = 200 rpm	Start: 212 ± 36 End: 242 ± 15	Start: 221 ± 27 End: 243 ± 27	Start: 0.43 End: 0.14
*H.* *polymorpha* RB11 P_FMD_-GFP	Syn6-MES medium, 10 g L^−1^ glycerol	30 °C V_L_ = 10 mL, n = 350 rpm,	452 ± 51	374 ± 27	0.66
*E.* *coli* BL21 (DE3) pRotHi-YFP	Wilms-MOPS medium, 20 g L^−1^ glucose, 0.5 g L^−1^ sorbitol	37 °C, V_L_ = 10 mL, n = 350 rpm	365 ± 21	370 ± 27	0.68
*E.* *coli* BL21 (DE3) pRotHi-YFP	Wilms-MOPS medium, 0.55 g L^−1^ glucose, 2 g L^−1^ lactose, 5 g L^−1^ glycerol	37 °C, V_L_ = 23 mL, n = 350 rpm	261 ± 34	200 ± 27	0.68

### Oxygen-unlimited cultivation of *H. polymorpha* RB11 P_FMD_-GFP with one initial carbon source

**Fig. 3:**
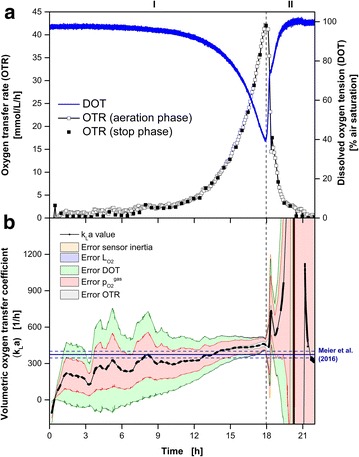
Oxygen-unlimited cultivation of *H.* *polymorpha* RB11 P_FMD_-GFP. **a** Simultaneous online monitoring of the oxygen transfer rate (OTR) measured with a RAMOS device and the dissolved oxygen tension (DOT) measured via dispersed oxygen-sensitive nanoparticles in the same RAMOS shake flask. **b** Calculation (Eq. 1) and error estimation (Eq. 2) of the k_L_a values during RAMOS aeration phases. The total error is represented by the surrounding area while the fraction of each error component is indicated by a *different color* as explained in the text. For comparison the k_L_a value (k_L_a_Meier_ = 374 h^−1^) estimated according to the correlation of Meier et al. [34] is shown by the* solid* and *dashed* (± 27 h^−1^) *dark blue lines*. The osmolality was assumed to be constant for the calculation of k_L_a_Meier_ and taken from the literature (*Osmol* = 0.66 Osmol kg^−1^). Cultivation conditions: Synthetic Syn6-MES medium with 10 g L^−1^ glycerol and 0.1 g L^−1^ oxygen-sensitive nanoparticles, 250 mL RAMOS shake flask, 30 °C, n = 350 rpm, V_L_ = 10 mL, shaking diameter d_0_ = 50 mm

Figure 3 shows the cultivation of *H. polymorpha* RB11 P_FMD_-GFP on buffered synthetic Syn-6-MES medium with 10 g L^−1^ glycerol as the initial carbon source. Based on the online measurements of the OTR and DOT, the cultivation can be divided into two phases: Exponential growth (I: 0–17.9 h) and the phase of decreasing respiratory activity (II: 17.9–22 h) [29, 35]. The end of exponential growth after 17.9 h due to carbon source depletion is indicated by a peak in the OTR curve (OTR = 42 mmol L^−1^ h^−1^) [7, 29, 35]. No oxygen limitation can be identified [11, 12]. Also by means of the online DOT signal, oxygen limitation can be excluded. The minimal DOT value occurred after 17.9 h at 39.3 % air saturation.

In Fig. 3b the calculated k_L_a values with their corresponding uncertainties according to Eqs. 1 and 2 are presented. The characteristics of the five considered errors were similar to the cultivation of *K.lactis*. The minimal overall error could be determined at the end of the exponential growth phase with a k_L_a_exp_ value of 452 ± 51 h^−1^. According to the empirical correlation of Meier et al. [34], the k_L_a_Meier_ value was 374 ± 27 h^−1^ (dark blue lines) for the applied cultivation conditions. Both values are compatible within their uncertainties.

### Oxygen-limited cultivation of *E. coli* BL21 (DE3) pRotHi-YFP with two initial carbon sources

**Fig. 4:**
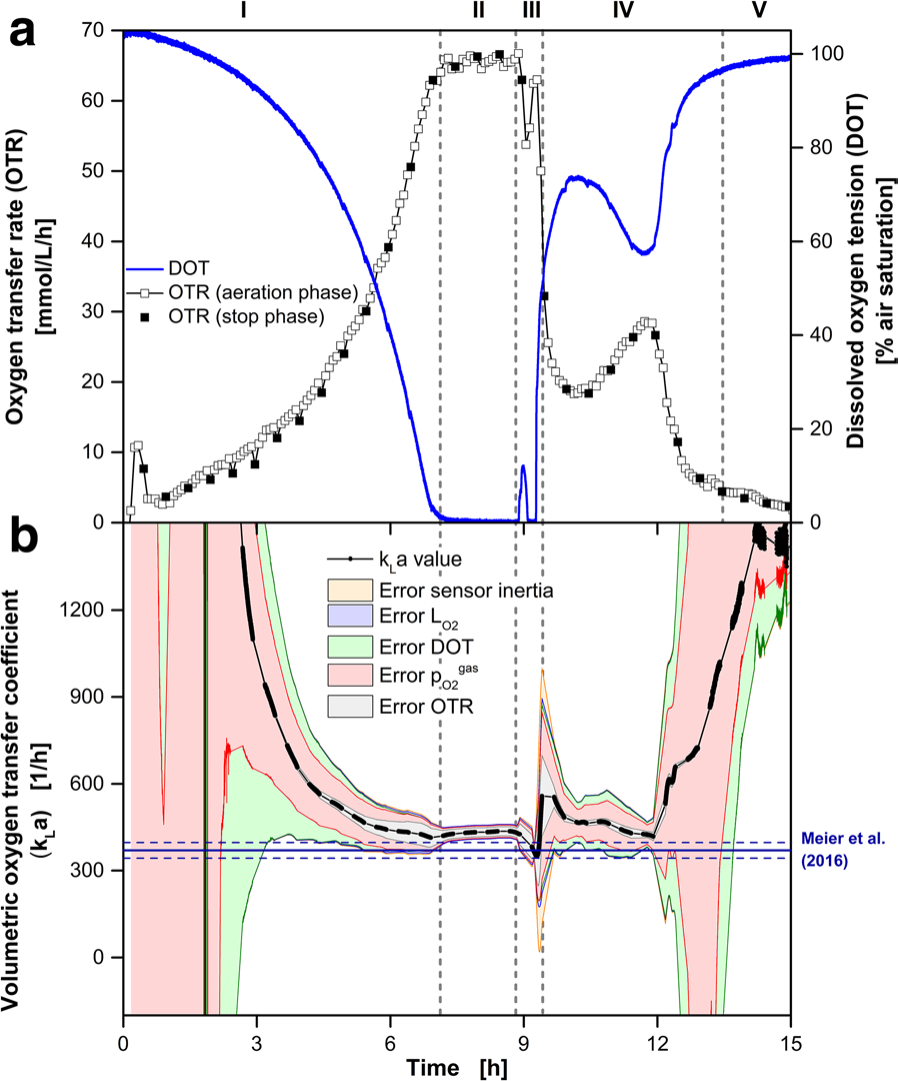
Oxygen-limited cultivation of *E. coli* BL21 (DE3) pRotHi-YFP. **a** Simultaneous online monitoring of the oxygen transfer rate (OTR) measured with a RAMOS device and the dissolved oxygen tension (DOT) measured via dispersed oxygen-sensitive nanoparticles in the same RAMOS shake flask. **b** Calculation (Eq. 1) and error estimation (Eq. 2) of the k_L_a values during RAMOS aeration phases. The total error is represented by the surrounding area while the fraction of each error component is indicated by a *different color* as explained in the text. For comparison the k_L_a value (k_L_a_Meier_ = 370 h^−1^) estimated according to the correlation of Meier et al. [34] is shown by the *solid and dashed* (± 27 h^−1^) *dark blue lines*. The osmolality was assumed to be constant for the calculation of k_L_a_Meier_ and taken from the literature (*Osmol* = 0.68 Osmol kg^−1^). Cultivation conditions: Synthetic Wilms-MOPS medium with 20 g L^−1^ glucose, 0.5 g L^−1^ sorbitol and 0.1 g L^−1^ oxygen-sensitive nanoparticles, 250 mL RAMOS shake flask, 37 °C, n = 350 rpm, V_L_ = 10 mL, shaking diameter d_0_ = 50 mm

Figure 4 presents the cultivation of *E. coli* BL21 (DE3) pRotHi-YFP on a synthetic medium with two initial carbon sources (20 g L^−1^ glucose, 0.5 g L^−1^ sorbitol) to challenge the applied measurement techniques. Hansen et al. [13] have already investigated this type of cultivation. Based on reported offline measurements in the literature [13] and the presented online signals (Fig. 4a), the cultivation can be divided into five phases. After exponential growth (I: 0–7.1 h) on the first initial carbon source glucose, oxygen limitation occurred (II: 7.1–8.8 h). During oxygen limitation, acetate was formed as overflow metabolite [13]. After glucose depletion, a metabolic switch to a growth on sorbitol as the second initial carbon source (III: 8.8–9.3 h) was indicated by peaks in the OTR and DOT curves at 9 h. Based merely on the OTR, it cannot be determined whether oxygen limitation was apparent during the short period of growth on sorbitol (III). Only by involving the DOT signal did it become certain that a second oxygen limitation appeared. A DOT close to 0 % air saturation was reached again (9.1–9.25 h). This indicates the shorter response time and resolution of the DOT than the OTR measurements. Even though the sorbitol concentration was rather low (0.5 g L^−1^), the metabolic switch could be distinctly determined with the applied DOT and OTR measurement techniques. After sorbitol depletion, a steep decrease of the OTR and an analogous steep increase of the DOT followed. A third growth phase on acetate, generated during the period of oxygen limitation, started (IV: 9.3–13.5 h). After 13.5 h, all carbon sources were depleted and the phase of decreasing respiratory activity (IV: 13.5–15 h) indicated the end of the cultivation.

In Fig. 4b the calculated k_L_a values with their corresponding uncertainties according to Eqs. 1 and 2 are shown. The characteristics of the five considered errors were similar to the previous cultivations. The minimal overall error could be determined during the first oxygen limitation (II), due to the reduced influence of the systematic errors of the $p_{{O_{2} }}^{gas}$ and DOT measurements. During this phase, the error of the k_L_a determination was dominated by the propagation of σ_OTR_ resulting in a k_L_a_exp_ value of 365 ± 21 h^−1^. According to the empirical correlation of Meier et al. [34], the k_L_a_Meier_ value was 370 ± 27 h^−1^ (dark blue lines) for the applied cultivation conditions. Both k_L_a values are well compatible within their uncertainties.

### Oxygen-unlimited cultivation of *E. coli* BL21 (DE3) pRotHi-YFP with three initial carbon sources

**Fig. 5:**
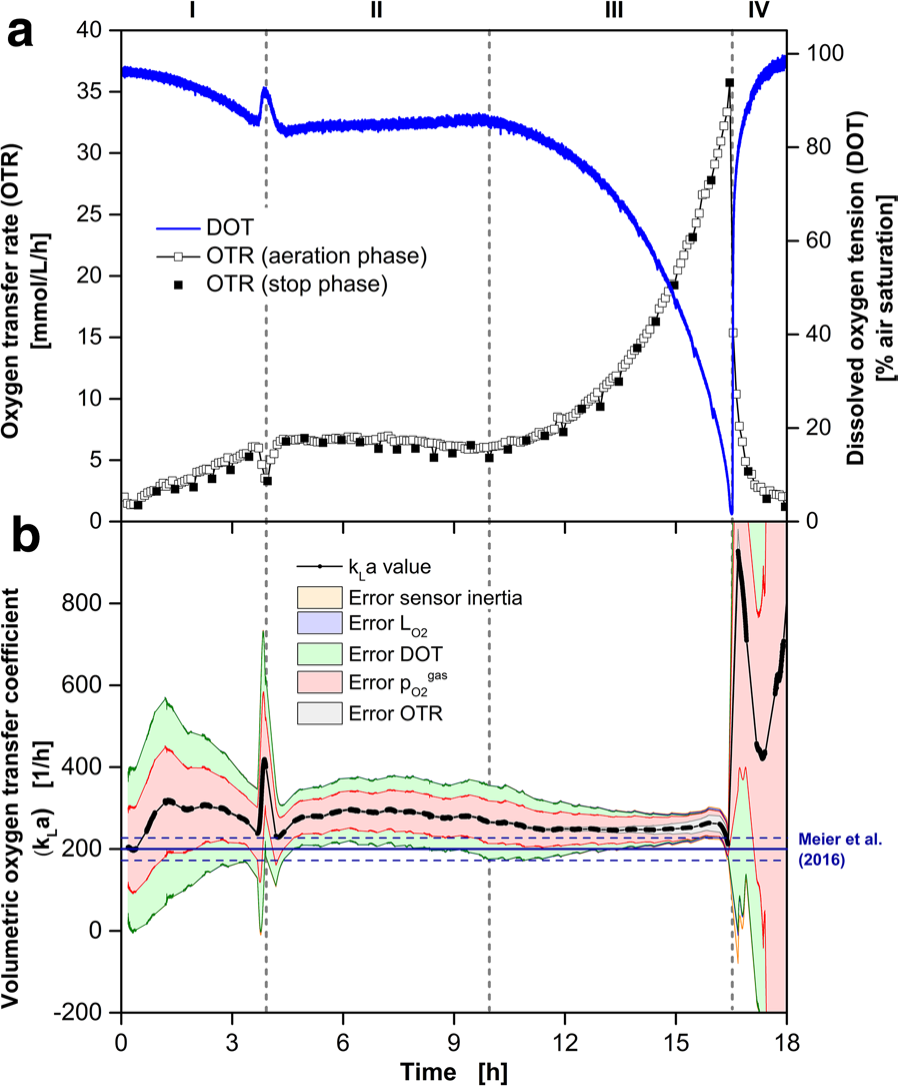
Oxygen-unlimited cultivation of *E.* *coli* BL21 (DE3) pRotHi-YFP. **a** Simultaneous online monitoring of the oxygen transfer rate (OTR) measured with a RAMOS device and the dissolved oxygen tension (DOT) measured via dispersed oxygen-sensitive nanoparticles in the same RAMOS shake flask. **b** Calculation (Eq. 1) and error estimation (Eq. 2) of the k_L_a values during RAMOS aeration phases. The total error is represented by the surrounding area while the fraction of each error component is indicated by a *different color* as explained in the text. For comparison the k_L_a value (k_L_a_Meier_ = 200 h^−1^) estimated according to the correlation of Meier et al. [34] is shown by the *solid and dashed* (± 27 h^−1^) *dark blue lines*. The osmolality was assumed to be constant for the calculation of k_L_a_Meier_ and taken from the literature (*Osmol* = 0.68 Osmol kg^−1^). Cultivation conditions: Synthetic Wilms-MOPS medium with 0.55 g L^−1^ glucose, 2 g L^−1^ lactose, 5 g L^−1^ glycerol and 0.1 g L^−1^ oxygen-sensitive nanoparticles, 250 mL RAMOS shake flask, 37 °C, n = 350 rpm, V_L_ = 23 mL, shaking diameter d_0_ = 50 mm

As a fourth example, a cultivation of *E.coli* BL21 (DE3) pRotHi-YFP in Wilms-MOPS auto-induction medium with three different initial carbon sources (0.55 g L^−1^ glucose, 2 g L^−1^ lactose, 5 g L^−1^ glycerol) is presented. Figure 5 shows the online measured DOT, OTR (Fig. 5a) and the corresponding determined k_L_a value (Fig. 5b). Based on the obtained online signals, the cultivation can be divided into four phases. Since no offline samples were analyzed, the carbon source concentration (glucose, lactose and glycerol), product fluorescence intensity and OTR during *E. coli* cultivation are given in Additional file 1 for a comparable strain based on data from Rahmen et al. [36]. During phase I (0–4 h) the first carbon source glucose was metabolized. The depletion is indicated by the peak in the OTR and DOT signal after 4 h. The Expression of yellow fluorescent protein (YFP) is induced by the consumption of lactose (II: 4–10 h, data not shown). From the literature, it is known that this *E.* *coli* strain consumes lactose and the third carbon source glycerol simultaneously during this phase [36, 37]. After the depletion of lactose (10 h), YFP expression stopped and enhanced growth on the remaining glycerol occurred (III: 10–16.5 h). This is indicated by the exponential OTR increase and the corresponding exponential DOT decrease. After 16.5 h, a maximum OTR (36 mmol L^−1^ h^−1^) was reached. By means of the DOT signal, the risk of oxygen limitation becomes obvious. At 16.5 h, a minimal DOT value of roughly 1.6 % air saturation was reached. Thus, glycerol was exhausted shortly before oxygen limitation (0 % air saturation) would have occurred. The phase of decreasing respiratory activity (IV) was entered after 16.5 h due to the absence of any further carbon source.

In Fig. 5b the calculated k_L_a values with their corresponding uncertainties according to Eqs. 1 and 2 are shown. The characteristics of the five considered errors are similar to the previous cultivations. The most accurate determination of the k_L_a value is possible at 16.5 h. At this time the influence of the otherwise dominant errors due to the DOT and $p_{{O_{2} }}^{gas}$ measurements was strongly reduced and the k_L_a_exp_ was determined as 261 ± 34 h^−1^. Under the applied cultivation conditions, a k_L_a_Meier_ value with an uncertainty of 200 ± 27 h^−1^ was predicted according to the correlation of Meier et al. [34]. Both values are compatible within their uncertainties.

## Conclusions

If fast-growing microorganisms like bacteria or yeast are cultivated, high shaking frequencies and low filling volumes are required to avoid oxygen limitation. Under these conditions, optical sensor spots in shake flasks provide erroneous values due to a mixed signal from the gas in the headspace of the flask and the DOT in the cultivation broth [18].

In this work, by using dispersed oxygen-sensitive nanoparticles, an easy to use, robust and reliable DOT measurement was developed, which is applicable for almost all cultivation conditions in shake flasks and for both, soluble complex and synthetic media. Reliable DOT measurements were achieved with 0.1 g L^−1^ oxygen-sensitive nanoparticles added to the cultivation broth. Media containing insoluble components were not investigated, but a possible applicability of the introduced technique by increasing the oxygen-sensitive nanoparticles concentration can be presumed. At fermenter scale, there is no need to apply oxygen-sensitive nanoparticles for DOT measurement. Classical electrochemical Clark electrodes or optical techniques based on optodes can be applied in large scale fermenters. Furthermore, the costs would be very high due to the necessary minimal concentration of nanoparticles at high volumes. But the introduced measurement system is a valuable alternative for existing DOT measurement systems in shake flask cultivations, especially at low filling volumes and high shaking frequencies. In combination with RAMOS data it was possible to determine k_L_a values online during cultivations of *K.lactis*, *E. coli* and *H. polymorpha*. The determined k_L_a values were investigated with respect to their corresponding uncertainties caused by systematic errors of OTR, DOT, $L_{{O_{2} }}$ and $p_{{O_{2} }}^{gas}$ estimations and sensor inertia. All k_L_a values were in good agreement with an empirical correlation based on the medium osmolality from the literature [34].

## Methods

### Microorganisms

In this study, three different microorganisms and four media were applied: *K. lactis* GG799 pKlac1 in complex YEP medium, *H. polymorpha* RB11 pC10-FMD P_FMD_-GFP in synthetic Syn-6-MES medium and *E. coli* BL21 (DE3) pRotHi-YFP in synthetic Wilms-MOPS medium containing glucose, lactose and glycerol, as well as synthetic Wilms-MOPS medium containing glucose and sorbitol, respectively. *K. lactis* GG799 pKlac1 was kindly provided by the Institute for Molecular Biotechnology of the RWTH Aachen University (Germany). *E. coli* BL21 (DE3) pRotHi YFP and *H. polymorpha* RB11 pC10-FMD PFMD-GFP was kindly was kindly provided by the Institute for Molecular Enzyme Technology (IMET) and the Institute for Microbiology at the Heinrich-Heine-University Düsseldorf (Germany).

### Media and cultivation

All pre-cultures were carried out in shake flasks. The cultivation vessels for the main cultures were 250 mL RAMOS shake flasks for the OTR, CTR, RQ and DOT measurements. The shaking diameter *d*_*0*_ was 50 mm for all cultivations. Every main culture medium for DOT measurements contained 0.1 g L^−1^ dispersed oxygen-sensitive nanoparticles (OXNANO, Pyro Science GmbH, Aachen, Germany). All main cultivations were inoculated with pre-cultures adjusted to a starting OD_600_ of 0.1. If not otherwise specified, all media and the 100-fold stock solution of the oxygen-sensitive nanoparticles were autoclaved for 20 min at 121 °C and 1 bar. All media reagents were of analytical grade and purchased from Carl Roth GmbH and Co. (Karlsruhe, Germany).

The complex YEP medium for the pre and main cultivation of *K.lactis* GG799 pKlac1 consisted of 10 g L^−1^ yeast extract (lot number: 180156693) and 20 g L^−1^ peptone/tryptone (lot number: 134209944). The pH was adjusted with 5M KOH to a value of 4.8. The pre-culture contained 20 g L^−1^ and the main culture 40 g L^−1^ glucose as carbon source. The pre-culture was inoculated with a 1 mL cryoculture containing 200 g L^−1^ glycerol. For pre and main cultivation the shaking frequency and filling volume were n = 200 rpm and V_L_ = 10 mL.

Synthetic Syn-6-MES medium for the pre- and main cultivation of *H.* *polymorpha* RB11 pC10-FMD P_FMD_-GFP was prepared according to the literature [29, 38]. The pre-cultivation was inoculated with a cryoculture (200 g L^−1^ glycerol stocks) and cultivated at 30 °C. A shaking frequency and filling volume of n = 350 rpm and V_L_ = 10 mL were applied.

For *E.coli* BL21 (DE3) pRotHi-YFP, two pre-cultivations were conducted according to the literature [29, 36]. For the first pre-cultivation terrific broth (TB) medium consisting of 12 g L^−1^ tryptone (lot number: 474216187), 24 g L^−1^ yeast extract (lot number: 180156693), 12.54 g L^−1^ K_2_HPO_4_, 2.31 g L^−1^ KH_2_PO_4_, and 5 g L^−1^ glycerol dissolved in water was used. The pH value was 7.2 ± 0.2 and not explicitly adjusted. The first pre-cultivations were inoculated with complex medium (TB) cryocultures (200 g L^−1^ glycerol stocks, OD = 1). Modified Wilms and Reuss medium (henceforth referred as Wilms-MOPS medium) [39] was used for the second pre-cultivation. It consisted of 20.9 g L^−1^ 3-(N-morpholino)-propanesulfonic acid (MOPS, 0.2 M), 20 g L^−1^ glucose, 5 g L^−1^ (NH_4_)_2_SO_4_, 3.0 g L^−1^ K_2_HPO_4_, 2 g L^−1^ Na_2_SO_4_, 0.5 g L^−1^ NH_4_Cl, 0.5 g L^−1^ MgSO_4_·7H_2_O, 0.01 g L^−1^ thiamine hydrochloride and 1 mL L^−1^ trace element solution. The trace element solution contained 41.76 g L^−1^ FeCl_3_·6H_2_O, 1.98 g L^−1^ CaCl_2_·2H_2_O, 0.54 g L^−1^ CoCl_2_·6H_2_O, 0.54 g L^−1^ ZnSO_4_·7H_2_O, 0.48 g L^−1^ CuSO_4_·5H_2_O, 0.3 g L^−1^ MnSO_4_·H_2_O and 33.39 g L^−1^ Na_2_EDTA (Titriplex III). The pH was adjusted with 5M NaOH to a value of 7. 50 mg L^−1^ sterile filtrated kanamycin was added to the medium. For the main cultivation, a modified Wilms-MOPS auto-induction medium was used. Compared to the medium of the second pre-culture, the 20 g L^−1^ glucose was replaced by 5 g L^−1^ glycerol, 2 g L^−1^ lactose and 0.55 g L^−1^ glucose.

For the second main cultivation of *E. coli* BL21 (DE3) pRotHi-YFP, the modified Wilms-MOPS medium of the second pre-cultivation was supplemented with 1.5 g L^−1^ sorbitol.

### Measurement setup

**Fig. 6:**
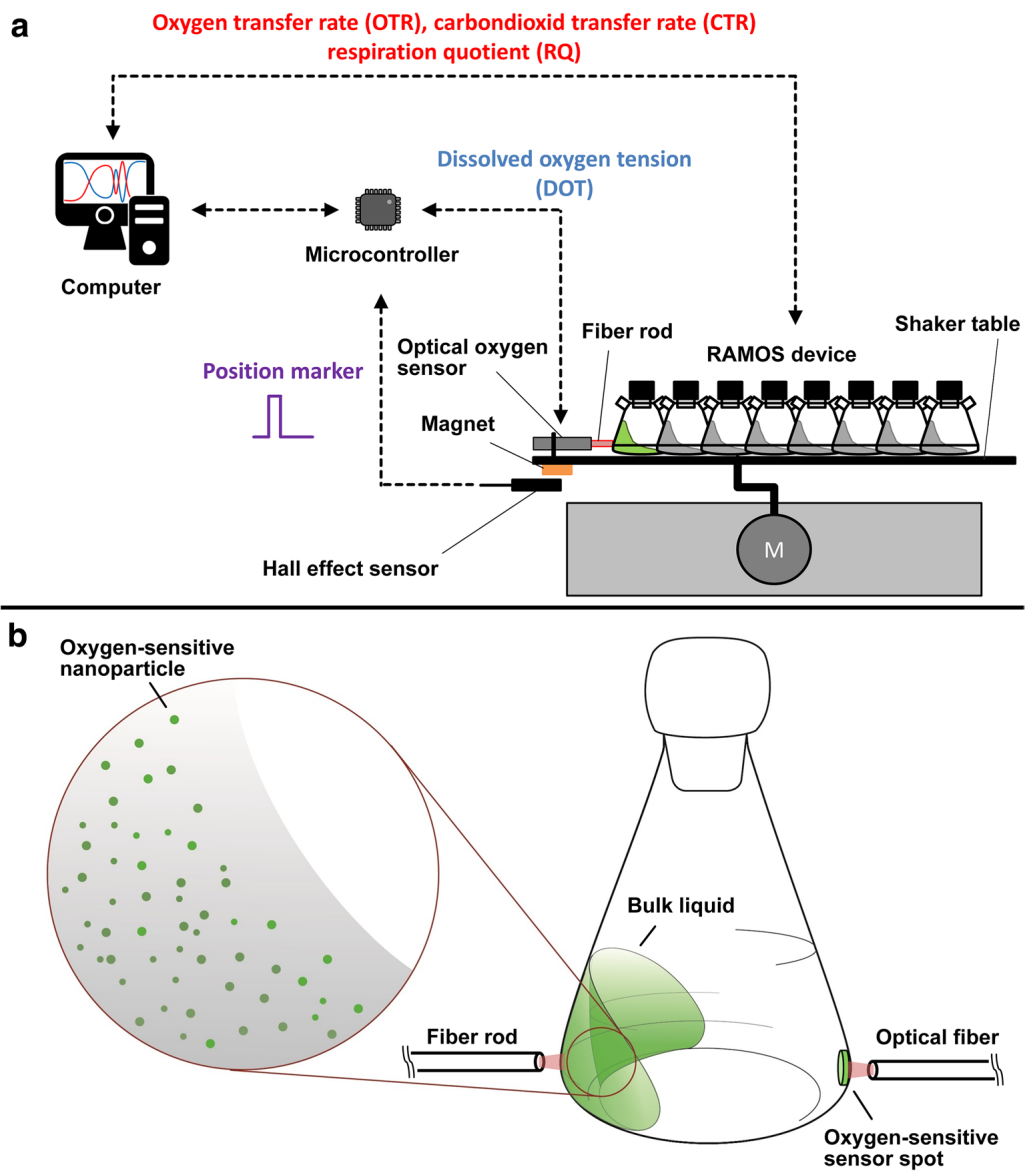
Measurement setup. **a** Experimental setup for simultaneous measurements of the dissolved oxygen tension (DOT) and respiration activity (OTR, CTR, RQ). The start of the DOT measurement is triggered by a hall effect sensor with respect to the position of the shaker table. **b** Setup for simultaneous DOT measurements via oxygen-sensitive nanoparticles and an oxygen-sensitive sensor spot (optode)

Figure 6a shows the experimental setup. A RAMOS device with eight shake flasks (built in-house according to Anderlei et al. [12]; electrochemical oxygen sensor: MAX-250 B, Maxtec Inc., Salt Lake City, USA) was located on an orbital shaker (shaking diameter: 50 mm) and controlled by a computer to obtain OTR, CTR and RQ. Additionally, the fiber rod (diameter: 3 mm) of an optical oxygen sensor (Piccolo2-OEM (P/N: PICO2-OEM), Pyro Science GmbH, Aachen, Germany) was mounted on the shaker table, facing one of the eight RAMOS shake flasks. Since only one shake flask was equipped with an optical oxygen sensor, only the DOT and the corresponding OTR are shown in Figs. 2, 3, 4 and 5. The OTR of up to 4 parallel cultivations with and without DOT monitoring were in good agreement and are not presented for clarity of presentation. To obtain a reliable and meaningful DOT signal, it is necessary to carry out each DOT measurement when the rotating bulk is at the same position in front of the fiber rod. Since the bulk liquid of non-viscous systems follows the shaker movement, the measurement can be triggered by a Hall effect sensor, which monitors the position of the shaker table. The optical oxygen sensor itself does not directly allow measurements triggered by an external pulse. Therefore, a microcontroller (Arduino Micro, Conrad Electronic SE, Hirschau, Germany) was interposed between the Hall effect sensor and optical oxygen sensor as a driver for the measurement. This setup allows a DOT measurement at specific time points, each triggered by the pulse of the hall effect sensor.

The overall measurement rate for the DOT measurement within the bulk liquid is approximately 1 Hz at a shaking frequency of 350 rpm. In preliminary tests, it was observed that the fluorescence intensity decreased roughly 5 % in a time period of 6 days (data not shown). The oxygen-sensitive nanoparticles are commercially available for €580 for 50 mg (11/2015) and, thus, a cost of €11.60 per cultivation with 10 mL filling volume needs to be taken into account. The DOT measurement technique was used in this work in a range of 0–100 % air saturation. This corresponds to an oxygen air content of 0 % to roughly 21 %. The manufacturer Pyro Science GmbH [33] states a possible applicability up to 50 % oxygen content in a gas, which corresponds to a DOT of roughly 240 % air saturation at standard conditions.

To compare the DOT measurement via oxygen-sensitive optodes and oxygen-sensitive nanoparticles, both techniques were utilized within one shake flask (Fig. 6b). The oxygen-sensitive optode (OXSP5, Pyro Science GmbH, Aachen, Germany) was glued (SCRINTEC, E046.1, Carl Roth GmbH, Karlsruhe, Germany) to the inner wall of the shake flask and sterilized by autoclaving (20 min, 121 °C). By means of an optical fiber with lens adapter (SPFIB-CL2 + SPADBAS, Pyro Science GmbH, Aachen, Germany), the optode-based DOT measurement was carried out with a commercial device (FireStingO2 (P/N: FSO2-4), Pyro Science GmbH, Aachen, Germany). Additionally, oxygen-sensitive nanoparticles (OXNANO, Pyro Science GmbH, Aachen, Germany) were dispersed within the liquid and utilized as described before.

Osmolality was measured by a cryoscopic osmometer (Osmomat 030, Gonotec GmbH, Berlin, Germany).

### k_L_a calculation

The k_L_a is calculated according to Eq. 1:

1 $${\text{k}}_{\text{L}} {\text{a}} = \frac{\text{OTR}}{{{\text{L}}_{{{\text{O}}_{2} }} \cdot \left( {{\text{p}}_{{{\text{O}}_{2} }}^{\text{gas}} - \frac{\text{DOT}}{100} \cdot {\text{p}}_{{{\text{O}}_{2} }}^{\text{cal}} } \right)}}$$

where $p_{{O_{2} }}^{gas}$ is the oxygen partial pressure [bar] in the headspace of the shake flask, $p_{{O_{2} }}^{cal}$ is the headspace oxygen partial pressure during calibration (0.21 bar), DOT is the dissolved oxygen tension of the cultivation broth [% air saturation] and $L_{{O_{2} }}$ is the oxygen solubility [mol L^−1^ bar^−1^]. $L_{{O_{2} }}$ was calculated according to the literature [40–42]; $p_{{O_{2} }}^{cal}$ and $L_{{O_{2} }}$ were considered as constants during the whole cultivation. The analysis method of Hansen et al. [13] was applied to expand the OTR information with OTR values within the aeration phases. Due to non-equilibrium conditions during the stop phases of the RAMOS device, the k_L_a calculation is only reasonable during aeration phases. For each $p_{{O_{2} }}^{gas}$ measurement point (every 2.88 s) of the aeration phases of the RAMOS device, an OTR and DOT value was interpolated to calculate a k_L_a value. For sensitivity tests of the k_L_a determinations, the well-known formula for Gaussian error propagation of independent variables was applied to Eq. 1. The resulting formula for the propagated error of the k_L_a value ($\sigma_{{k_{L} a}}$) is shown by Eq. 2.

2 $$\varvec{\sigma}_{{\varvec{k}_{\varvec{L}} \varvec{a}}}^{2} = \left( {\frac{1}{{\varvec{L}_{{\varvec{O}_{2} }} \cdot \left( {\varvec{p}_{{\varvec{O}_{2} }}^{{\varvec{gas}}} - \frac{{\varvec{DOT}}}{100} \cdot \varvec{p}_{{\varvec{O}_{2} }}^{{\varvec{cal}}} } \right)}}} \right)^{2} \cdot\varvec{\sigma}_{{\varvec{OTR}}}^{2} + \left( {\frac{{\varvec{OTR}}}{{\varvec{L}_{{\varvec{O}_{2} }} \cdot \varvec{ }\left( {\varvec{p}_{{\varvec{O}_{2} }}^{{\varvec{gas}}} - \frac{{\varvec{DOT}}}{100} \cdot \varvec{p}_{{\varvec{O}_{2} }}^{{\varvec{cal}}} } \right)^{2} }}} \right)^{2} \cdot\varvec{\sigma}_{{\varvec{p}_{{\varvec{O}_{2} }}^{{\varvec{gas}}} }}^{2} + \left( {\frac{{\varvec{OTR}}}{{\varvec{L}_{{\varvec{O}_{2} }}^{2} \cdot \varvec{ }\left( {\varvec{p}_{{\varvec{O}_{2} }}^{{\varvec{gas}}} - \frac{{\varvec{DOT}}}{100} \cdot \varvec{p}_{{\varvec{O}_{2} }}^{{\varvec{cal}}} } \right)}}} \right)^{2} \cdot\varvec{\sigma}_{{\varvec{L}_{{\varvec{O}_{2} }} }}^{2} + \varvec{ }\left( {\frac{{\varvec{OTR} \cdot \frac{{\varvec{p}_{{\varvec{O}_{2} }}^{{\varvec{cal}}} }}{100}}}{{\varvec{L}_{{\varvec{O}_{2} }} \cdot \varvec{ }\left( {\varvec{p}_{{\varvec{O}_{2} }}^{{\varvec{gas}}} - \frac{{\varvec{DOT}}}{100} \cdot \varvec{p}_{{\varvec{O}_{2} }}^{{\varvec{cal}}} } \right)^{2} }}} \right)^{2} \cdot\varvec{\sigma}_{{\varvec{DOT}}}^{2}$$

where σ_OTR_ [mmol L^−1^ h^−1^] is the assumed systematic error of the measured *OTR* (5 %), ${{\sigma }}_{{{\text{p}}_{{{\text{O}}_{2} }}^{\text{gas}} }}$ [bar] is the assumed systematic error of the measured $p_{{O_{2} }}^{gas}$ (3 %), ${{\sigma }}_{{{\text{L}}_{{{\text{O}}_{2} }} }}$ [mol L^−1^ bar^−1^] is the assumed systematic error of the calculated $L_{{O_{2} }}$ (2 %) and σ_DOT_ [% air saturation] is the assumed systematic error of the measured *DOT* (3 %). An overview of the assumptions for the error estimation is given in Table 2.

**Table 2:** Summary of assumed systematic errors for k_L_a calculations based on the measured DOT and OTR

Term	Assumed systemic error (%)	Unit
*σ*_*OTR*_	5	mol L^−1^ h^−1^
$\sigma_{{p_{{O_{2} }}^{gas} }}$	3	bar
*σ*_*DOT*_	3	% air saturation
$\sigma_{{L_{{O_{2} }} }}$	2	mol L^−1^ bar^−1^

Based on this error analysis it was possible to estimate an error for each k_L_a value. Due to the different measurement principles, the chemical sensor for $p_{{O_{2} }}^{gas}$ measurements and the optical DOT sensor differed in their response time. To estimate the error propagating on the k_L_a value due to this different behavior, the DOT values were shifted for plus and minus one minute. The difference between the resulting and the original k_L_a values were considered as “sensor inertia” and are presented in Figs. 2, 3, 4 and 5.

## Abbreviations

DOT: dissolved oxygen tension (% air saturation)
GFP: green fluorescent protein
OTR: oxygen transfer rate (mol L^−1^ h^−1^)
RQ: respiratory quotient
RAMOS: respiration activity monitoring system
YFP: yellow fluorescent protein

### List of symbols

d_0_: shaking diameter [mm]
DOT: dissolved oxygen tension (% air saturation) k_L_aVolumetric oxygen transfer coefficient (h^−1^)
L_O2_: oxygen solubility (mol L^−1^ bar)
$$p_{{O_{2} }}^{gas}$$
oxygen partial pressure in gas phase (bar)
$$p_{{O_{2} }}^{cal}$$
oxygen partial pressure in gas phase during calibration (bar)
V_L_: liquid filling volume (mL)
$$\sigma_{{k_{L} a}}$$
error of k_L_a estimation (h^−1^)
*σ*_*OTR*_: assumed systematic error of OTR estimation (mol L^−1^ h^−1^)
$$\sigma_{{p_{{O_{2} }}^{gas} }}$$
assumed systematic error of $p_{{O_{2} }}^{gas}$ estimation (bar)
*σ*_*DOT*_: assumed systematic error of *DOT* estimation (% air saturation)
$$\sigma_{{L_{{O_{2} }} }}$$
assumed systematic error of $L_{{O_{2} }}$ estimation (mol L^−1^ bar^−1^)

## Additional file

10.1186/s12934-016-0444-4 Oxygen transfer rate (OTR), product (FbFP) fluorescence intensity and carbon source concentrations (glycerol, lactose and glucose) during oxygen-unlimited cultivation of *E. coli* BL21 (DE3): pET22b(+)–His6-LOV-BSLA-Lys170Glu. Four cultivation phases (I-IV) are identified by the OTR curve: (I) increase in OTR due to growth on glucose until glucose depletion; (II) constant OTR due to product (FbFP) formation during parallel growth on lactose and glycerol. The end of this phase corresponds to the depletion of lactose (10.7 h); (III) increase in OTR (13.5 h, 31 mmol L^-1^h^-1^) due to growth on residual glycerol; (IV) end of cultivation after depletion of glycerol as last carbon source. Cultivation conditions: Synthetic Wilms-MOPS medium with 0.5 g L^-1^ glucose, 2 g L^-1^ lactose, 5 g L^-1^ glycerol, 250 mL RAMOS shake flask, 37 °C, n = 350 rpm, V_L_ = 10 mL, shaking diameter d_0_ = 50 mm. The presented data has already been published by Rahmen et al. [36].
